# Chitosan Coating as a Strategy to Increase Postemergent Herbicidal Efficiency and Alter the Interaction of Nanoatrazine with *Bidens pilosa* Plants

**DOI:** 10.1021/acsami.4c03800

**Published:** 2024-07-12

**Authors:** Bruno T. Sousa, Lucas B. Carvalho, Ana C. Preisler, Telma Saraiva-Santos, Jhones L. Oliveira, Waldiceu A. Verri, Giliardi Dalazen, Leonardo F. Fraceto, Halley Oliveira

**Affiliations:** † Department of Agronomy, State University of Londrina (UEL), 86057-970 Londrina, Paraná, Brazil; ‡ Institute of Science and Technology, São Paulo State University (UNESP), 18087-180 Sorocaba, São Paulo, Brazil; § Department of Pathology, State University of Londrina (UEL), 86057-970 Londrina, Paraná, Brazil; ∥ Department of Animal and Plant Biology and Department of Agronomy, State University of Londrina (UEL), 86057-970 Londrina, Paraná, Brazil

**Keywords:** Biopolymers, PSII Inhibition, Nanocarriers, Nanopesticides, Surface Charge, Nanoparticle Uptake, Weed Control

## Abstract

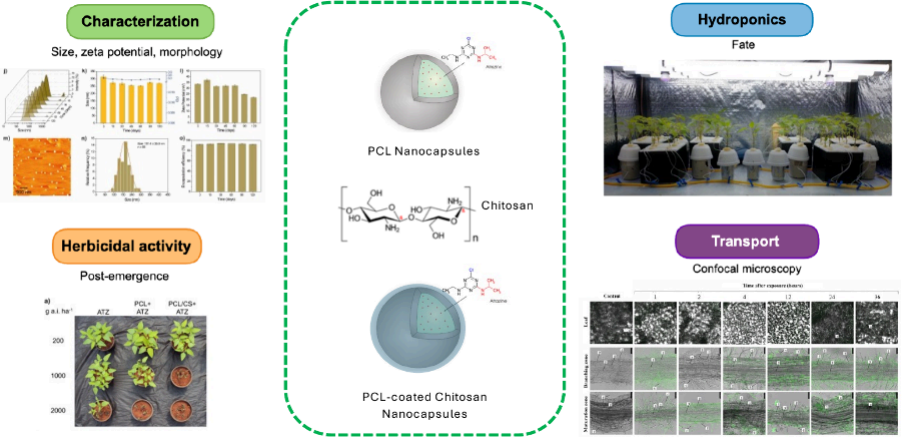

The atrazine nanodelivery system, composed of poly(ε-caprolactone) (PCL+ATZ) nanocapsules (NCs), has demonstrated efficient delivery of the active ingredient to target plants in previous studies, leading to greater herbicide effectiveness than conventional formulations. Established nanosystems can be enhanced or modified to generate new biological activity patterns. Therefore, this study aimed to evaluate the effect of chitosan coating of PCL+ATZ NCs on herbicidal activity and interaction mechanisms with *Bidens pilosa* plants. Chitosan-coated NCs (PCL/CS+ATZ) were synthesized and characterized for size, zeta potential, polydispersity, and encapsulation efficiency. Herbicidal efficiency was assessed in postemergence greenhouse trials, comparing the effects of PCL/CS+ATZ NCs (coated), PCL+ATZ NCs (uncoated), and conventional atrazine (ATZ) on photosystem II (PSII) activity and weed control. Using a hydroponic system, we evaluated the root absorption and shoot translocation of fluorescently labeled NCs. PCL/CS+ATZ presented a positive zeta potential (25 mV), a size of 200 nm, and an efficiency of atrazine encapsulation higher than 90%. The postemergent herbicidal activity assay showed an efficiency gain of PSII activity inhibition of up to 58% compared to ATZ and PCL+ATZ at 96 h postapplication. The evaluation of weed control 14 days after application ratified the positive effect of chitosan coating on herbicidal activity, as the application of PCL/CS+ATZ at 1000 g of a.i. ha^–1^ resulted in better control than ATZ at 2000 g of a.i. ha^–1^ and PCL+ATZ at 1000 g of a.i. ha^–1^. In the hydroponic experiment, chitosan-coated NCs labeled with a fluorescent probe accumulated in the root cortex, with a small quantity reaching the vascular cylinder and leaves up to 72 h after exposure. This behavior resulted in lower leaf atrazine levels and PSII inhibition than ATZ. In summary, chitosan coating of nanoatrazine improved the herbicidal activity against *B. pilosa* plants when applied to the leaves but negatively affected the root-to-shoot translocation of the herbicide. This study opens avenues for further investigations to improve and modify established nanosystems, paving the way for developing novel biological activity patterns.

## 1 Introduction

The use of herbicides for weed management is a common practice in agriculture. Synthetic molecules of various chemical groups are designed to promote the death of weeds and increase crop productivity.^1,2^ Identifying new herbicides is a time-consuming and expensive process, so the correct use and conservation of existing herbicide molecules is necessary.^1,2^ Nanoencapsulation of herbicides is a strategy to encapsulate herbicide molecules without altering their mechanism of action, providing new physicochemical properties to formulations that are superior to the conventional properties. These include increases in the active ingredient absorption and delivery efficiency and reductions in application losses, environmental impacts, and water and energy consumption, due to less frequent application.^3−7^

Atrazine is an herbicide molecule frequently used in maize, sorghum, and sugarcane cultivation.^8,9^ It belongs to the triazine chemical group and inhibits photosystem II (PSII), making it suitable for use in control both pre-emergence and early postemergence of dicotyledonous weeds and some grasses.^1,8^ However, atrazine presents drawbacks, including high persistence in soils and frequent contamination of natural aquatic environments due to its large-scale use.^8,10,11^

The application of nanotechnology in developing carrier systems for active ingredients allows for more efficient and safer agricultural practices, with fewer side effects.^12,13^ The carrier system using nanocapsules (NCs) of poly(ε-caprolactone) for atrazine (PCL+ATZ) has shown high efficiency in delivering the active ingredient to target plants, yielding promising results for weed control in pre- and postemergence assays.^14−17^ Application in postemergence has demonstrated increased PSII inhibition efficiency in species like *Alternathera tenella*, *Amaranthus viridis*, *Bidens pilosa*, *Digitaria insularis*, and *Raphanus raphanistrum*.^14,16−18^ These gains are attributed to improved characteristics of the nanoformulation, such as increased leaf adhesion, faster penetration into the leaf mesophyll through natural leaf apertures (such as hydathodes and stomata), and increased translocation to younger plant tissues.^17,19^

Additionally, it has been demonstrated that the herbicidal activity of atrazine via root absorption is potentiated by PCL+ATZ NCs, reaching the vascular cylinder of the roots and being transported to the above-ground parts of the plants.^20^ This characteristic enhances the herbicidal activity of the nanosystem in pre-emergent weed control, as previously reported by Preisler.^15^ In *Lactuca sativa* plants, the application of PCL+ATZ NCs in the substrate increased oxidative stress parameters in comparison with the conventional formulation at the same dose.^21^ Moreover, PCL NCs themselves were toxic to *Brassica juncea* seeds, reducing germination and increasing the number of abnormal seedlings.^22^

The coating of NCs is a strategy to reduce phytotoxic effects and/or alter their physicochemical properties. For PCL NCs, a potential coating option is chitosan, a natural, biodegradable, and biocompatible biopolymer derived from chitin, the second most abundant carbon-containing polymer on Earth.^23−25^ Its biocompatibility can reduce the phytotoxicity of active ingredients when encapsulated and of nanoparticles when coated.^25^ Chitosan is cationic, capable of changing the zeta potential of nanoparticles (from negative to positive) through interactions with surface electric charges.^24,26^ This nanoparticle coating strategy has shown promising results for drug delivery due to its adhesive properties.^27,28^

Interactions between NCs and plants (absorption, transport, accumulation, and transformation) need further clarification to aid in the development of the most efficient systems for delivering active ingredients.^7^ It is known that nanoparticle adhesion, absorption, and translocation depend on the electrical potential and other characteristics (such as size, shape, and polymer matrix), with the xylem being a crucial pathway for nanoparticle distribution in plants.^7,29−31^ Changes in the surface charge of nanoparticles can confer new biological activity patterns,^32^ and these are yet to be evaluated in plants for the formulation of PCL+ATZ NCs after chitosan coating. Therefore, the objective of the current study was to characterize the chitosan-coated PCL+ATZ NCs formulation regarding size, zeta potential, and polydispersity, as well as to evaluate its postemergent herbicidal activity and its absorption and translocation dynamics in *B. pilosa* plants through evaluations of PSII activity, weed control, endogenous levels of active ingredient, and in situ localization of fluorescently labeled NCs in plant tissues.

## 2 Materials and Methods

### 2.1 Synthesis of Nanoformulations

Poly(ε-caprolactone) (PCL) NCs were prepared using the antisolvent nanoprecipitation method.^33^ An organic phase was prepared by dissolving 100 mg of PCL, 40 mg of sorbitan monostearate surfactant, and 200 mg of caprylic/capric acid triglyceride in 30 mL of acetone. For the formulation containing atrazine, 10 mg of atrazine was added to this organic phase. The organic phase was introduced into 30 mL of an aqueous phase, consisting of a 0.2% (w/v) solution of polysorbate 80 surfactant. After 20 min of orbital stirring, the formulation was concentrated by using a rotary evaporator to a final volume of 10 mL. A control NC was obtained without the addition of atrazine (PCL) and an NC containing atrazine (PCL+ATZ) at 1 g of a.i. L^–1^.

For the coating of NCs, a chitosan solution was prepared at 0.5% (w/v) in a 1% (v/v) aqueous solution of acetic acid. The solubilization was performed overnight and filtered under vacuum conditions to eliminate any insoluble impurities. The nanoformulations were initially concentrated to 5 mL, and then 5 mL of the 0.5% chitosan solution was slowly added under constant stirring (which was maintained for 1 h after addition of the chitosan solution). The following formulations were obtained: coated NC control without the addition of the active ingredient (PCL/CS) and coated NCs containing atrazine (PCL/CS+ATZ) at 1 g of a.i. L^–1^.

### 2.2 Functionalization of Chitosan with FITC and Preparation of Labeled Nanoformulations

The synthesis of FITC-labeled chitosan was carried out following the methodology described by Colonna.^34^ Low molecular weight chitosan (2.5 g) was dissolved in 250 mL of 0.1 mol L^–1^ acetic acid, followed by the addition of 250 mL of methanol and 125 mL of a methanolic solution of FITC (500 mg L^–1^). The reaction was conducted for 3 h in the dark under constant stirring. Subsequently, the labeled chitosan was precipitated by adding 0.5 mol L^–1^ NaOH until pH 10 was reached. The precipitate was recovered by centrifugation at 1700*g* for 15 min. An exhaustive washing process was performed by resuspending the precipitate in a methanol/water solution (70:30, v/v), followed by centrifugation, until absorbance signals (in the UV–vis range) were no longer observed in the supernatant. The labeled chitosan was then dissolved in 0.1 mol L^–1^ acetic acid and dialyzed in the dark against ultrapure water for 5 days, with two daily water changes during dialysis. Finally, the FITC-labeled chitosan was obtained by lyophilization. The labeled chitosan was characterized by FTIR through transmittance measurements obtained by the diffuse reflection method on KBr pellets containing 1% (w/w) of the sample. Jasco FTIR-410 infrared equipment was used, operating in the range of 400–4000 cm^–1^, with 64 scans and a resolution of 8 cm^–1^.

For the preparation of the labeled nanoformulation, the steps were followed as described earlier, using a proportion of 30% FITC-functionalized chitosan mixed with unlabeled chitosan. The coating of NCs was also carried out as described previously, resulting in labeled NCs containing atrazine (PCL/CS*f*+ATZ) at 1 g of a.i. L^–1^.

### 2.3 Characterization and Stability of Nanoformulations

The hydrodynamic size and polydispersity index (PDI) of the NCs were determined by dynamic light scattering (DLS), while microelectrophoresis was employed to measure the zeta potential. The colloidal suspensions of PCL, PCL+ATZ, PCL/CS, and PCL/CS+ATZ were diluted in ultrapure water (2:1000, v/v) for these measurements and analyzed with a Zetasizer Nano ZS 90 instrument (Malvern) operated at a 90° detection angle. The measurements were conducted in triplicate at 25 °C over a storage period of 120 days.

The suspension of PCL/CS*f*+ATZ was characterized byusing nanoparticle tracking analysis (NTA) with a NanoSight LM 10 cell (532 nm) equipped with a CMOS camera and NanoSight software (version 3.2). The nanocapsule suspension was diluted in deionized water (1:20000, v/v), and five injections were made at a temperature of 25 °C, with 60 s videos collected for each injection.

The morphology and size analysis of PCL/CS+ATZ NCs were performed with atomic force microscopy (AFM). A diluted suspension of NCs in ultrapure water (1:30000, v/v) was drop-cast onto a silicon plate and dried in a desiccator. Micrographs were obtained with an Easy Scan 2 instrument (Basic AFM – Standard BT02217; Nanosurf, Switzerland) operating in noncontact mode with a TapAl-G cantilever (BudgetSensor, Bulgaria) and a scanning rate of 90 Hz. Images were processed with the use of Gwyddion software.

The encapsulation efficiency of atrazine in PCL/CS NCs was indirectly determined by the ultrafiltration/centrifugation method. The atrazine concentration was quantified by using an ultra-high-performance liquid chromatography (UHPLC) Thermo Scientific Ultimate 3000 instrument with a Phenomenex Luna 5 μm C18 column (100 Å, 250 mm × 4.6 mm) maintained at 30 °C. The UV–vis detector operated at 223 nm. The mobile phase consisted of acetonitrile:water (50:50, v/v) with a flow rate of 1.5 mL min^–1^. The method’s limit of detection (LOD) was 1.19 μg L^–1^, and the limit of quantification (LOQ) was 3.96 μg L^–1^ (*R*^2^ = 0.9997). Triplicate measurements were performed over 120 days.

### 2.4 Release Assays

The release profile of atrazine from PCL/CS+ATZ was determined through release kinetic assays. A system with a donor and acceptor compartment, separated by a semipermeable cellulose membrane with a molecular exclusion pore of 1 kDa (SpectraPore), was set up to meet the sink condition. A volume of 495 μL of PCL/CS+ATZ or ATZ (Primóleo SC, 400 g of a.i. L^–1^, Syngenta) at 1 g of a.i. L^–1^ was added to the donor compartment. The membrane’s cross-sectional area (1.54 cm^2^) was immersed in the acceptor medium, consisting of 150 mL of an aqueous solution of polysorbate 80 (0.25% w/v). The system was maintained under constant agitation, and the released amount was monitored for 96 h. The assays were conducted in triplicate, and the released atrazine was quantified by UHPLC according to eq 1.
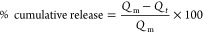
1where *Q*_m_ is the total amount of atrazine present in the formulation, and *Q*_*t*_ is the amount of atrazine released as a function of time.

The release data were fitted (*Q*_*t*_/*Q*_∞_ < 0.6) to the semiempirical Korsmeyer–Peppas kinetic model (eq 2).
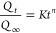
2where *Q*_∞_ corresponds to the total atrazine released at infinite time, *K* is the constant involving the structure and geometry of the system, and *n* is the exponent indicative of the herbicide release mechanism, valid for Fickian and non-Fickian diffusion processes.

### 2.5 Herbicidal Activity Assay

The herbicidal activity assay in the greenhouse involved experimental units comprising plastic pots with a 1 L capacity (10.5 cm height, 9.5 cm lower diameter, 14 cm upper diameter) filled with Eutric Nitisol clayey latosol soil collected from the Experimental Farm of the State University of Londrina (UEL). The soil had a high clay content typical for northern Paraná, with chemical characteristics as described: pH (CaCl_2_), 4.83; organic matter, 28.2 g dm^–3^; P, 7.63 mg dm^–3^; K, 0.65 cmol_c_ dm^–3^; Na, 0.0 cmol_c_ dm^–3^; Ca, 3.96 cmol_c_ dm^–3^; Mg, 1.80 cmol_c_ dm^–3^; sum of bases, 6.41 cmol_c_ dm^–3^; cation exchange capacity at pH 7.0 (CEC), 11.0 cmol_c_ dm^–3^; and base saturation (BS), 58.2%. Base saturation was calculated using eq 3.
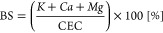
3

Each experimental unit contained five *B. pilosa* L. (Asteraceae) plants directly sown into the soil in the pots, with seeds collected from random plants on the UEL campus. The experiment followed a completely randomized design organized in a factorial scheme (3 × 3 + 1) with three formulations (ATZ, PCL+ATZ, and PCL/CS+ATZ) and three doses (200, 1000, and 2000 g of a.i. ha^–1^), each with four replications, plus an additional control treatment (only water). Formulations were applied between 7:00 AM and 8:30 AM via foliar spray with a manual sprayer at a volume of 5.1 mL per experimental unit.

Nondestructive physiological assessments of the maximum quantum efficiency of PSII (*F*_v_/*F*_m_) and the relative electron transport rate (rETR) were conducted at 24, 48, 72, and 96 h postapplication. A portable fluorometer model OS 1p (Opti-Sciences, Hudson, USA) was used for assessments. For *F*_v_/*F*_m_ data acquisition, plants required dark acclimation for 20 min. For rETR determination, the effective quantum efficiency of PSII (Δ*F*/*F*_m_′) was measured with plants acclimated to ambient light, monitored by a digital lux meter model LX1010B (Politerm, São Paulo, Brazil). *F*_v_/*F*_m_ measurements and rETR calculations were carried out following the procedure described by Sousa et al.^16^ Inhibition rates of PSII and rETR were calculated with the use of eqs 4 and 5.
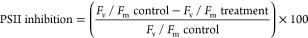
4
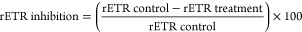
5

Weed control was assessed 14 days after application using the ALAM^35^ scale, assigning scores from 1 to 6 based on the percentage of control, as described: 1 = 0–40% control (poor, none); 2 = 41–60% (fair); 3 = 61–70% (sufficient); 4 = 71–80% (good); 5 = 81–90% (very good); and 6 = 91–100% (excellent).

After the control assessment, plants were collected and stratified into above-ground and root parts. Each portion of plant material was placed in paper bags (separated by experimental unit) and dried at 60 °C for 5 days. Following this period, the plant material was weighed with a semianalytical scale, RC 2013 (Sauter, Germany). For both above-ground and root portions, the mass reduction in relation to the control treatment was calculated with eq 6.
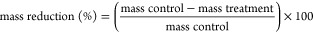
6

### 2.6 Absorption and Translocation Assay in Hydroponic System

Seeds of *B. pilosa* (from the same batch as the herbicide activity experiment) were sown in soil-filled pots. When the plants exhibited two pairs of fully expanded true leaves, they were transferred to a hydroponic system with the nutrient solution detailed in Table S1. The experiment took place in a laboratory, using aquariums with 2 L of nutrient solution, constant aeration, and a 12/12 h light/dark photoperiod. Before starting the treatments, the nutrient solution was replaced with distilled water, and the formulations ATZ, PCL+ATZ, or PCL/CS+ATZ were added to achieve a concentration of 8 mg of a.i. L^–1^. An additional treatment (control) was maintained with plants in water only. Each treatment had eight replicates. Nondestructive physiological assessments of the maximum quantum efficiency of PSII (*F*_v_/*F*_m_) and the relative electron transport rate (rETR) were performed at 2, 4, 8, 12, 24, 36, 48, and 72 h after exposure in hydroponics, using the methodology described in section 2.5.

### 2.7 Confocal Microscopy

The confocal microscopy assay was conducted with adaptations from Preisler et al.^20^ Tests were performed with *B. pilosa* plants grown in a hydroponic system similar to that used in the absorption and translocation assay. However, the evaluated treatments were the control (water) and PCL/CS+ATZ labeled with a fluorescent probe. To monitor the labeled NCs, the root system (main and lateral roots) and the second fully expanded youngest leaf were collected at exposure intervals of 1, 2, 4, 12, 24, and 36 h.

Samples were fixed for 4 h with 4% paraformaldehyde (PFA) at 4 °C, protected from light, and washed for 10 min in phosphate-buffered saline (PBS, pH 7.2). Segments (1 cm) from two root regions (maturation zone near the root apex and branching zone near the collar) and the midregion of the leaf, close to the central vein, were prepared for slide mounting using Fluormount (Southern Biotech). The samples were analyzed with a 20× objective on a Leica TCS SP8 confocal spectral microscope (Leica Microsystems, Wetzla, Germany) with excitation at 448 nm and emission from 457 to 558 nm (color range from blue to green).

As a negative control for parameter standardization and removal of all markers, plants not exposed to labeled NCs (treated with water) were used. Once the intensity and laser parameters of the negative control were standardized, they were applied to the treatments with labeled NCs. Therefore, the fluorescence observed in the images resulted from the NCs and not from constitutive elements, as it did not appear in the negative control. Bright-field microscopy was used to determine the region of interest and capture images. The images were processed, and fluorescence intensity was quantified using Leica LAS X LS software (version 3.5.7).

### 2.8 Extraction and Quantification of Atrazine and Fluorescent Probe in Plant Tissues

The extraction of FITC-labeled NCs from plant tissues was performed following the methodology of Carvalho et al.^36^ with some modifications. In summary, fresh plant tissues (roots, stems, and leaves) were incubated with 5 mL of NaOH at 70 °C for 60 min. The samples were then centrifuged for 15 min at 1700*g*. After a 10-fold dilution of the supernatant, the fluorescence was measured using a microplate reader (Tecan Infinite 200 Pro; Männedorf, Switzerland) with an excitation wavelength of 457 nm and an emission wavelength of 522 nm. The results were calculated by subtracting the average fluorescence signal of the control from the plant samples that did not receive the FITC probe treatment. The method’s detection limit was 125.3 ng L^–1^ with a quantification limit of 417.8 ng L^–1^ (*R*^2^ = 0.9996).

Plant tissues underwent a simultaneous two-stage extraction process for 24 and 36 h to determine the endogenous levels of atrazine. The process involved keeping the samples in conical tubes containing 10 mL of methanol with moderate agitation at 25 °C. The samples were centrifuged at 1700*g*, and the solid phase was subjected to the extraction process again. The methanolic extract obtained in the two stages was placed in amber vials without a cap for solvent evaporation and concentration to a final volume of nearly 5 mL. The samples were filtered through 0.22 μm nylon filters (Allcrom, São Paulo, Brazil), and atrazine was quantified by UHPLC. The analytical conditions were identical to those described for the determination of encapsulation efficiency (section 2.3).

### 2.9 Statistical Analysis

The inhibition data obtained in percentages were transformed by the arcsine square root transformation (√*x*). All data were tested for the normality of errors and homogeneity of variances. The herbicidal activity data in the greenhouse were subjected to a two-factor ANOVA using the *F* test (*p* ≤ 0.05), and when significant, means were compared using the Tukey test (*p* ≤ 0.05). For the results of confocal microscopy analyses and herbicidal activity in hydroponics, one-way ANOVA was used. When significant, the means were compared by the Tukey test (*p* ≤ 0.05).

## 3 Results

### 3.1 Characterization and Stability of Nanoformulations

**Figure 1:**
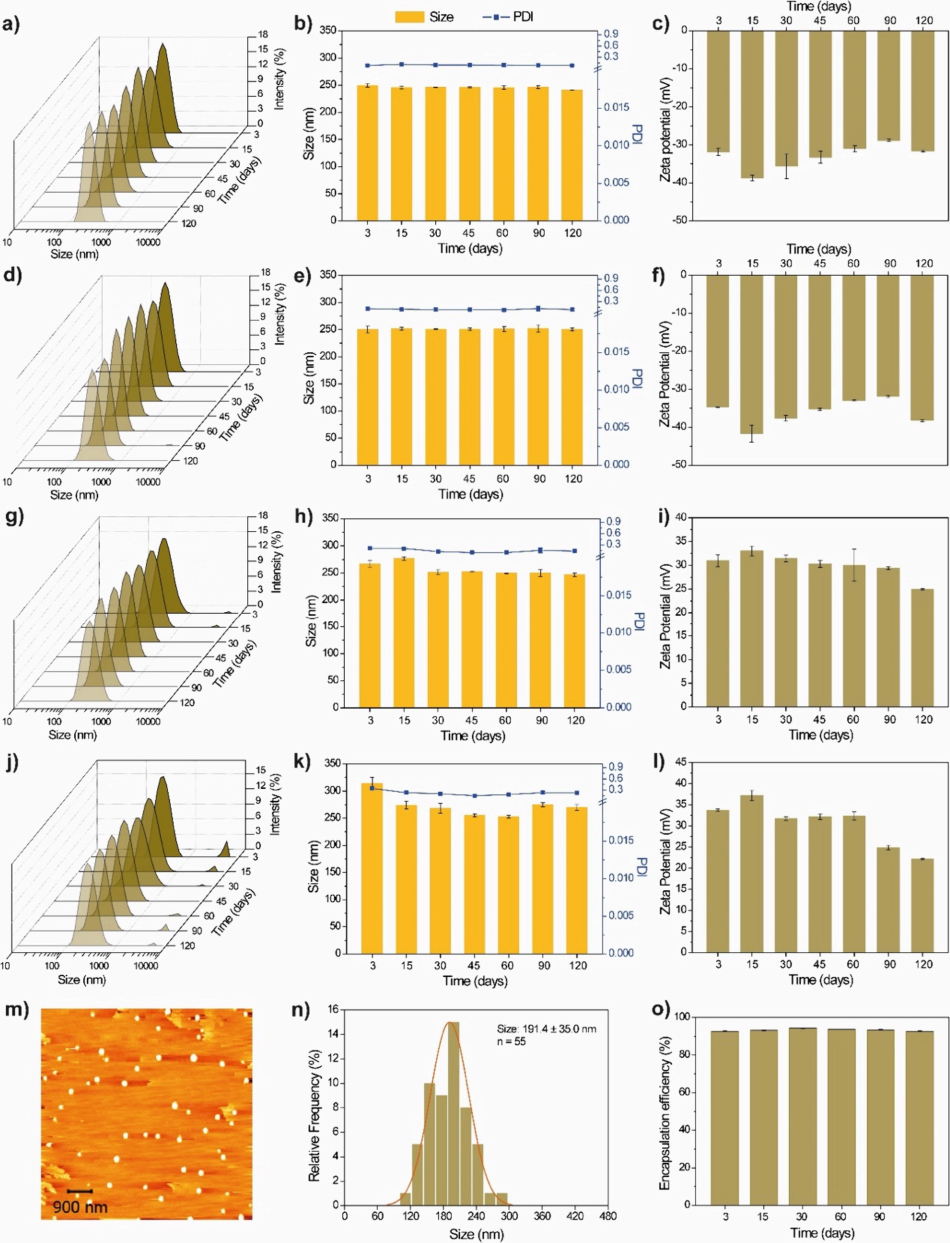
Size distribution, average hydrodynamic size, polydispersity index (PDI), and zeta potential for the nanoformulations: (a–c) control polycaprolactone (PCL) nanocapsules; (d–f) polycaprolactone nanocapsules containing atrazine (PCL-ATZ); (g–i) control polycaprolactone nanocapsules coated with chitosan (PCL/CS); (j–l) polycaprolactone nanocapsules containing atrazine and coated with chitosan (PCL/CS+ATZ); (m) morphology and (n) size distribution of PCL/CS+ATZ nanocapsules; (o) encapsulation efficiency of atrazine in PCL/CS+ATZ nanocapsules for a period of 120 days.

The PCL and PCL+ATZ NCs presented negative zeta potentials, and coating with chitosan made the PCL/CS and PCL/CS+ATZ NCs positively charged (ζ = 25 ± 2 mV), indicating the modification of the surface of these NCs. The systems remained stable during the monitoring period (120 days) (Figure 1), with an average size of PCL/CS+ATZ NCs of 262 ± 3, 193 ± 5, and 191 ± 35 nm, as determined by the DLS, NTA, and AFM techniques, respectively (the measurements correspond to the average size, specifically the peaks of the DLS analyses) (Figure 1). The system is predominantly monodispersed (PDI = 0.208 ± 0.03), the nanocapsule concentration remained in the range of 1012–1013 nanocapsules mL^–1^, and the encapsulation efficiency was above 90% during the analyzed period.

It is noteworthy that the functionalization of chitosan with the FTIC probe was successful, as demonstrated by FTIR analyses (Figure S1 and Table S2), showing shifts in the chitosan bands and the appearance of N=C=O bonds from the aromatic groups of fluorescein. PCL/CS*f*+ATZ presented a size similar to those of the nonlabeled formulations, with a diameter of approximately 194 nm and a nanoparticle concentration in the order of 1012, as shown in the NTA analyses (Figure S2).

**Figure 2:**
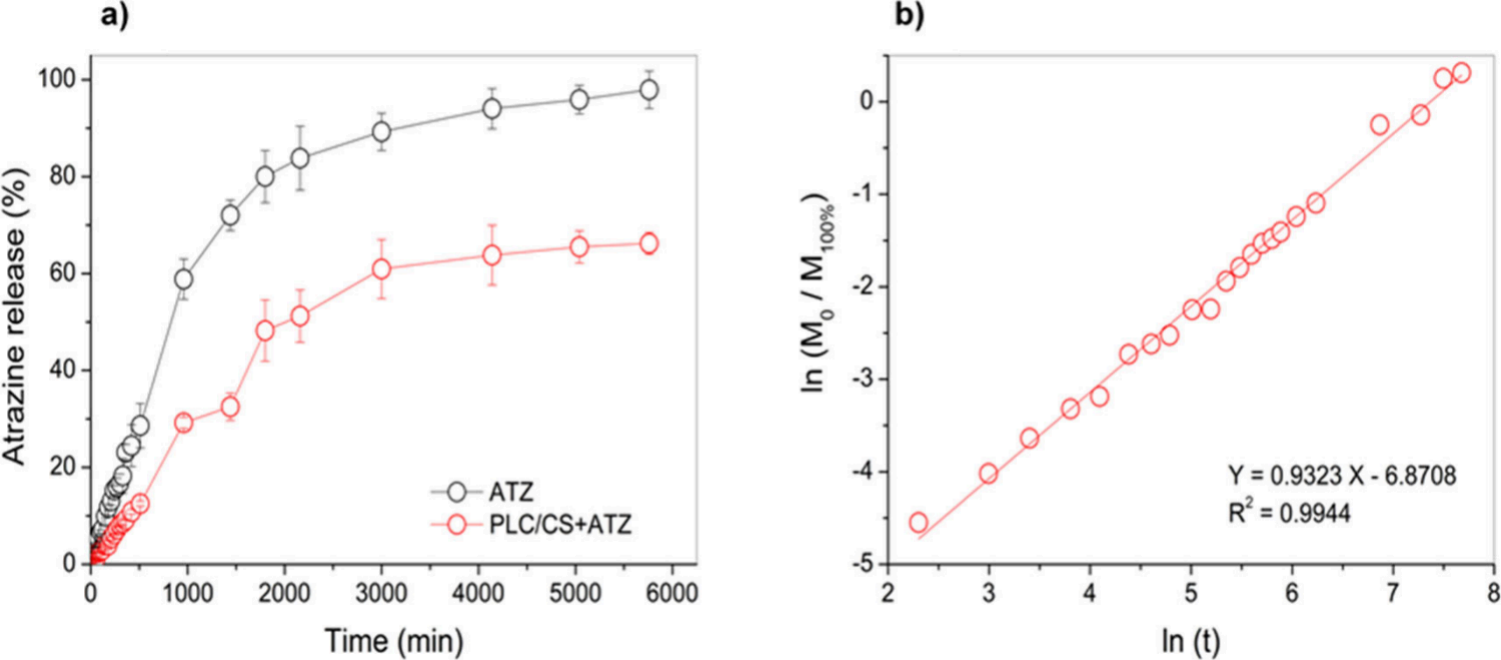
(a) Release curves for commercial atrazine (ATZ) and polycaprolactone nanocapsule nanoformulation containing atrazine and coated with chitosan (PCL/CS+ATZ). (b) Release data fitted to the Korsmeyer and Peppas kinetic model.

The release kinetics of atrazine, as obtained in in vitro assays through dialysis using a semipermeable membrane (Figure 2a), revealed alterations in the release profile of atrazine from the nanoformulation PCL/CS+ATZ. Nanoencapsulation led to a reduction in the atrazine release rate, with the released quantity being approximately 30% lower than that of the commercial herbicide after 92 h of the assay and about 60% lower within the first 24 h. The release data for PCL/CS+ATZ were fitted to the mathematical model of Korsmeyer–Peppas (0.9944) (Figure 2b), indicating that the release rate is governed by the swelling and relaxation of the polymeric matrix of the nanocapsules.^37,38^

### 3.2 Herbicidal Activity Evaluation

**Figure 3:**
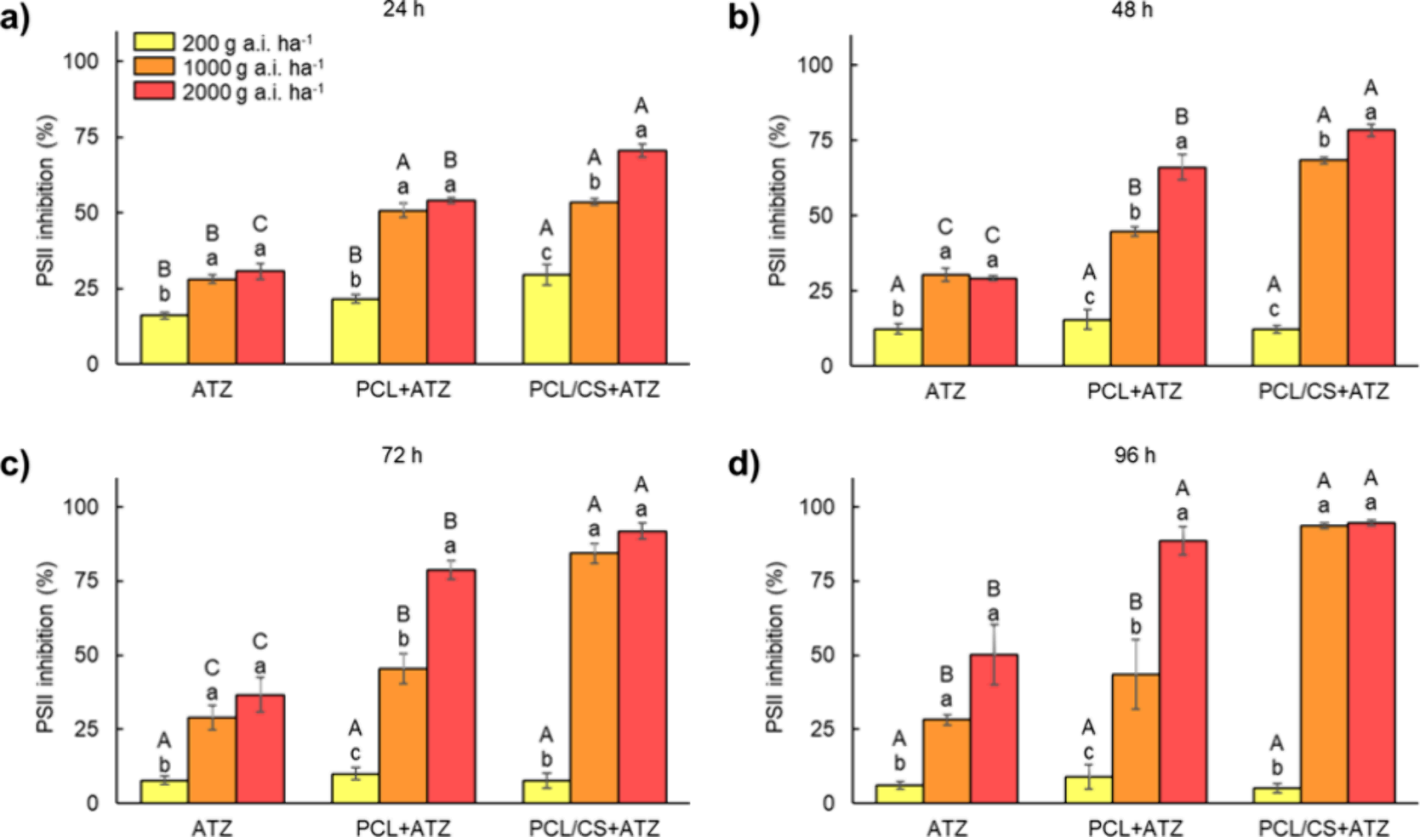
Inhibition of photosystem II (PSII) activity of *B. pilosa* plants at 24 (a), 48 (b), 72 (c), and 96 h (d) after foliar application of commercial atrazine (ATZ), polycaprolactone nanocapsules containing atrazine (PCL+ATZ), and atrazine-containing polycaprolactone nanocapsules coated with chitosan (PCL/CS+ATZ) in three doses of active ingredient (200, 1000, and 2000 g of a.i. ha^–1^). Different uppercase letters indicate differences between formulations within the same dose, and different lowercase letters indicate differences between doses of the same formulation by Tukey’s test (*p* ≤ 0.05). Data represent means ± standard deviation (*n* = 5).

The nanoatrazine formulation PCL/CS+ATZ provided a 70% inhibition of the maximum quantum yield of the photosystem II (PSII) in *B. pilosa* plants at 2000 g of a.i. ha^–1^, just 24 h after foliar application (Figure 3a). The inhibition percentage of this nanoformulation was 23.4% higher than that of PCL+ATZ and 39.7% higher than that of ATZ at the same dose. For 200 g of a.i. ha^–1^, the PCL/CS+ATZ formulation also exhibited the highest inhibition among the three formulations (29.7%), with a gain of 8% over PCL+ATZ and 13.5% over ATZ. At 1000 g of a.i. ha^–1^, the inhibitions provided by PCL/CS+ATZ and PCL+ATZ were similar and, on average, 24.1% higher than that of ATZ.

At 48 h after application, the PSII inhibitions resulting from the application of PCL/CS+ATZ at 1000 and 2000 g of a.i. ha^–1^ were 68.4 and 78.3%, respectively. Compared to the PCL+ATZ formulation, these represented gains of 23.7 and 12.2%, and compared to ATZ, the gains were 37 and 49.1% at the respective doses. However, the PCL+ATZ formulation still achieved gains in PSII inhibition over ATZ, which were 14.3 and 38% at 1000 and 2000 g of a.i. ha^–1^. No differences among formulations were observed for PSII inhibition at 200 g of a.i. ha^–1^.

At 72 h (Figure 3c), the highest PSII inhibition percentages continued to be provided by PCL/CS+ATZ, with 84.4 and 91.9% inhibition at 1000 and 2000 g of a.i. ha^–1^. These percentages represented efficiency gains of 38.9 and 13.1% over PCL+ATZ and 55.4 and 55.2% over ATZ, at the respective doses. The PCL+ATZ formulation also provided higher PSII inhibition than ATZ, by 16.5 and 42.2%, at 1000 and 2000 g of a.i. ha^–1^, respectively. At 96 h after application (Figure 3d), the PSII inhibition percentages at 1000 and 2000 g of a.i. ha^–1^ were above 90% for PCL/CS+ATZ. The PSII inhibitions provided by the PCL/CS+ATZ and PCL+ATZ formulations at the 2000 g of a.i. ha^–1^ dose were similar and, on average, 42% higher than that provided by ATZ. However, at the 1000 g of a.i. ha^–1^ dose, the PSII inhibitions provided by the PCL+ATZ and ATZ formulations were similar, averaging 57.9% lower than the PCL/CS+ATZ formulation.

**Figure 4:**
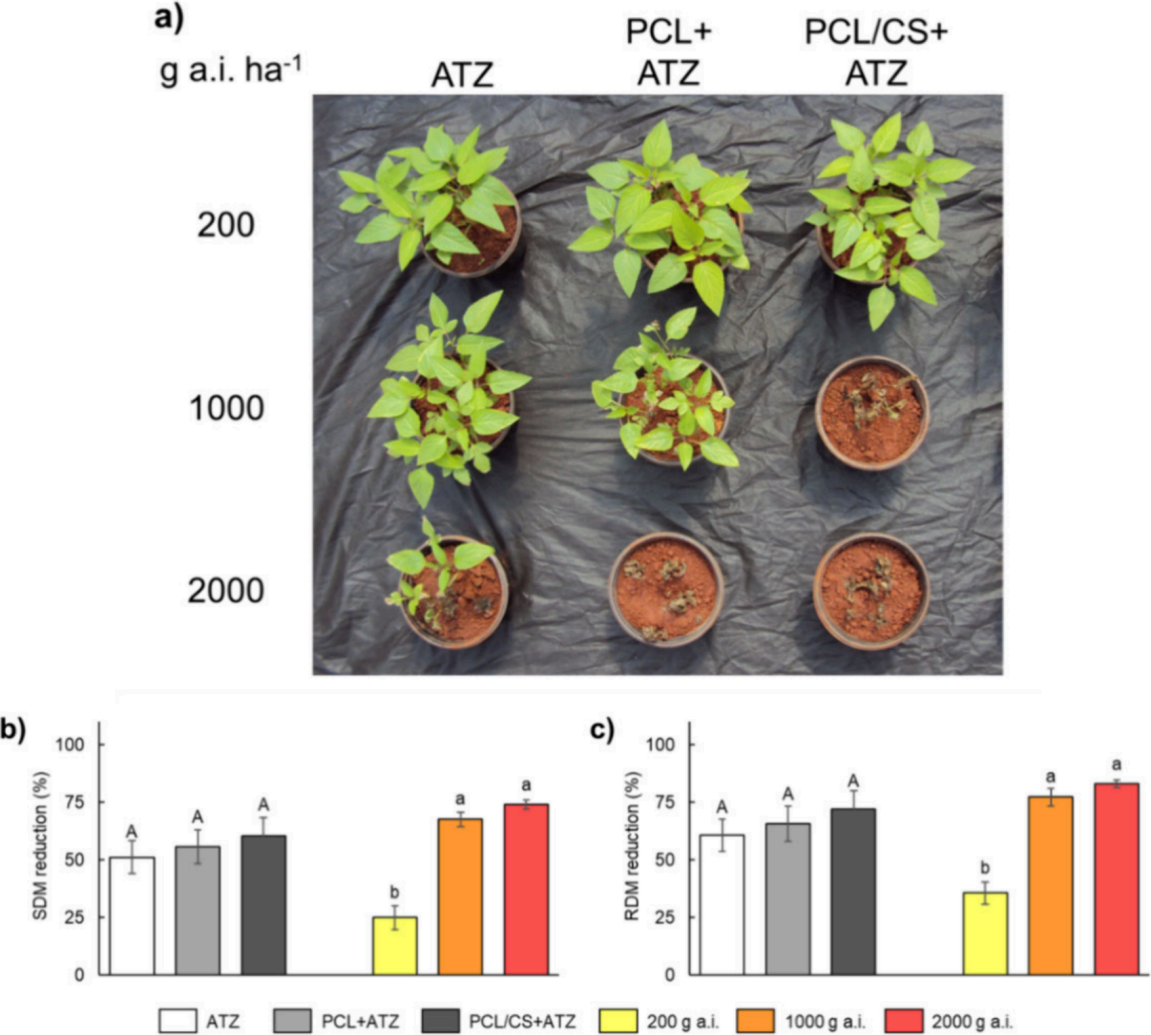
(a) Images of representative units, (b) shoot dry mass (SDM) reduction, and (c) root dry mass (RDM) reduction of *B. pilosa* plants 14 days after application of commercial atrazine (ATZ), polycaprolactone nanocapsules containing atrazine (PCL+ATZ), and atrazine-containing polycaprolactone nanocapsules coated with chitosan (PCL/CS+ATZ) in three doses of active ingredient (200, 1000, and 2000 g of a.i. ha^–1^). Different uppercase letters indicate differences between formulations regardless of dose, and different lowercase letters indicate differences between doses regardless of formulation by the Tukey test (*p* ≤ 0.05). Data represent means ± standard deviation (*n* = 5).

The visual assessment of *B. pilosa* control 14 days after foliar application demonstrated that the 200 g of a.i. ha^–1^ dose of any formulation was not sufficient to kill the plants (Table 1 and Figure 4a). However, with 1000 g of a.i. ha^–1^ of PCL/CS+ATZ, weed control was satisfactorily better than with 2000 g of a.i. ha^–1^ of ATZ, allowing a 50% reduction in the active ingredient dose without compromising atrazine’s herbicidal activity. At 200 g of a.i. ha^–1^, all three formulations received a score of 1, indicating poor or no plant control. Plant control scores were similar between ATZ and PCL+ATZ (score 2) at 1000 g of a.i. ha^–1^, while PCL/CS+ATZ scored 5 at the same dose. For plant control at 2000 g of a.i. ha^–1^, the PCL+ATZ and PCL/CS+ATZ formulations showed very good (score 5) and excellent (score 6) performances, while the performance of ATZ was good (score 4).

**Table 1:** Visual Evaluation Scores of Control of *B. pilosa* Plants 14 days after Application of Atrazine at Doses of 200, 1000, and 2000 g of a.i. ha^–1^ in the Formulations Conventional (ATZ), Polycaprolactone Nanocapsules (PCL+ATZ), and Polycaprolactone Nanocapsules Coated with Chitosan (PCL/CS+ATZ)^a^

g of a.i. ha^–1^	ATZ	PCL+ATZ	PCL/CS+ATZ
200	1	1	1
1000	2	2	5
2000	4	5	6

a ALAM scale (1974), grades: 1 = 0–40% control (poor, none); 2 = 41–60% (regular); 3 = 61–70% (sufficient); 4 = 71–80% (good); 5 = 81–90% (very good); 6 = 91–100% (excellent). Source: The author.

Despite the gains in PSII inhibition efficiency and weed control provided by the PCL/CS+ATZ and PCL+ATZ formulations, no differences were observed among formulations for the reductions in shoot and root dry masses (Figure 4b,c). However, higher percentages of reductions in dry masses were observed with doses of 1000 and 2000 g of a.i. ha^–1^ compared to 200 g of a.i. ha^–1^, regardless of the formulation.

### 3.3 Evaluation of Absorption and Transport of Nanocapsules in Hydroponic System

**Figure 5:**
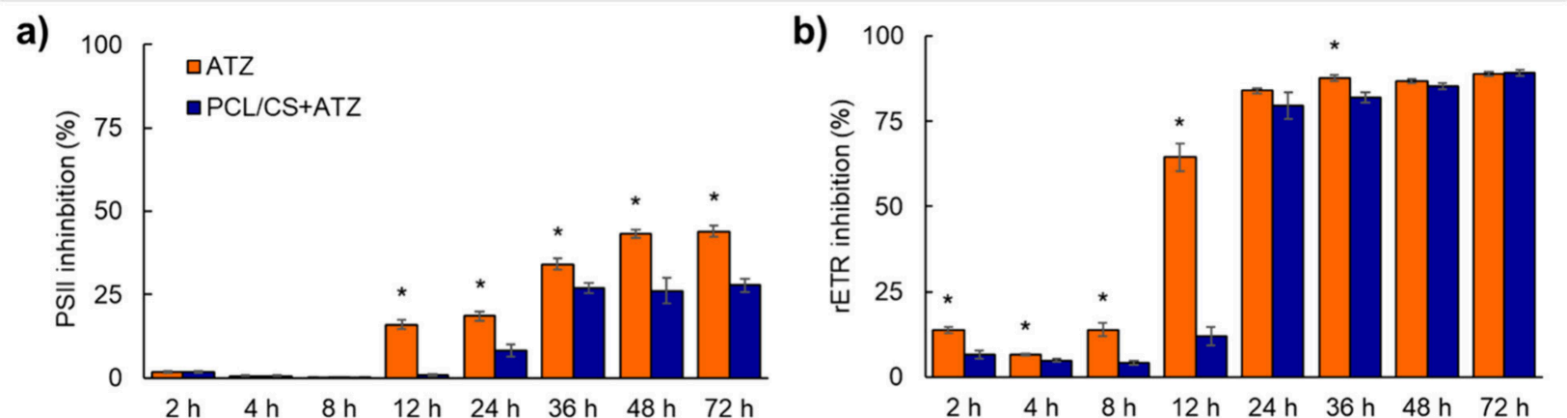
(a) Inhibition of photosystem II (PSII) activity and (b) inhibition of the relative electron transport rate (rETR) of *B. pilosa* plants exposed to atrazine (8 mg L^–1^) in hydroponic system with conventional formulation (ATZ) or polycaprolactone nanocapsules coated with chitosan (PCL/CS+ATZ). The asterisk (∗) indicates a significant difference between the PCL/CS+ATZ and ATZ treatments within each time point according to the *F* test (*p* ≤ 0.05). Data represent means ± standard deviation (*n* = 8).

After 2 and 8 h of exposure of *B. pilosa* plants to atrazine, minimal percentages of PSII inhibition occurred for both formulations (≈0.8%), showing no significant differences (Figure 5a). However, from 12 to 72 h of exposure, the lowest percentages of PSII inhibition were observed in plants exposed to PCL/CS+ATZ, ranging from 8.2 to 27.2%. The PSII inhibitions provided by PCL/CS+ATZ were, on average, 12.7% lower than those of ATZ (which reached 44% inhibition at 72 h). The inhibitions of rETR in *B. pilosa* plants exposed to ATZ at 2, 4, and 8 h were, on average, 7.4, 2, and 10% higher than those exposed to PCL/CS+ATZ (Figure 5b). At 12 h after exposure, the rETR inhibition caused by ATZ was 52.5% higher than that caused by PCL/CS+ATZ. From 24 h of exposure onward, the percentages of rETR inhibition induced by both formulations were similar, except at 36 h, when the rETR inhibition of ATZ was 5.7% higher than that of PCL/CS+ATZ.

**Figure 6:**
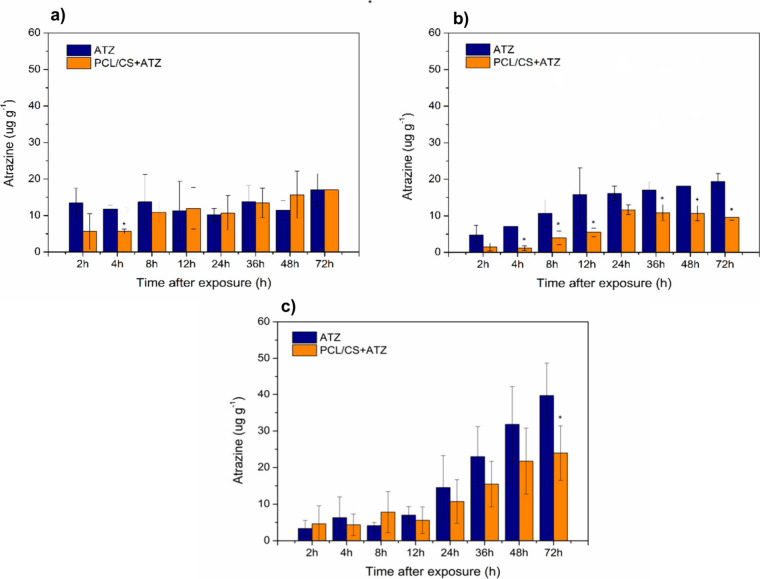
Quantification of endogenous atrazine levels in (a) roots, (b) stems, and (c) leaves of *B. pilosa* plants exposed to atrazine (8 mg L^–1^) in a hydroponic system with conventional formulation (ATZ) or atrazine-containing polycaprolactone nanocapsules coated with chitosan (PCL/CS+ATZ). The asterisk (∗) indicates a significant difference between the PCL/CS+ATZ and ATZ treatments within each time point according to the *F* test (*p* ≤ 0.05). Data represent means ± standard deviation (*n* = 5).

**Figure 7:**
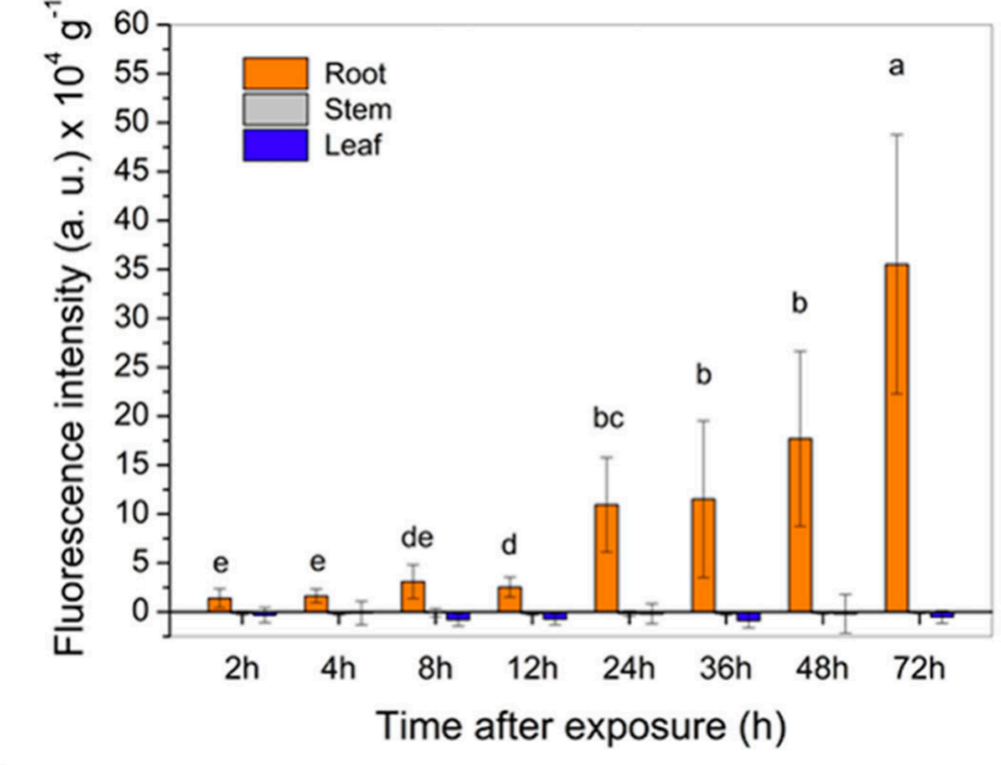
Fluorescence intensity measured in root, stem, and leaf extracts of *B. pilosa* plants hydroponically exposed to the atrazine-containing polycaprolactone nanocapsules coated with fluorescently labeled chitosan (PCL/CS*f*+ATZ nanoformulation). Different lowercase letters indicate differences among time points according to Tukey’s test (*p* ≤ 0.05). Data represent means ± standard deviation (*n* = 5.).

The endogenous atrazine levels were quantified in organs of hydroponically grown *B. pilosa* plants exposed to different formulations (Figure 6). In the roots, the herbicide levels remained unchanged in the ATZ treatment, while an increase in atrazine content over time was observed for PCL/CS+ATZ (Figure 6a). In the stem, an increase in atrazine concentration was observed until 12 h in the ATZ treatment and until 24 h for the nanoformulation (Figure 6b). In the leaves, the commercial formulation induced greater levels of atrazine in a shorter period of time than PCL/CS+ATZ (Figure 6c). Hydroponically grown *B. pilosa* plants were also exposed to the FITC-labeled nanoformulation (PCL/CS*f*+ATZ). The fluorescence intensity in roots increased over time, which clearly demonstrates the uptake and accumulation of NCs in this organ (Figure 7). However, the fluorescence signal was not detected in the stem or leaves.

**Figure 8:**
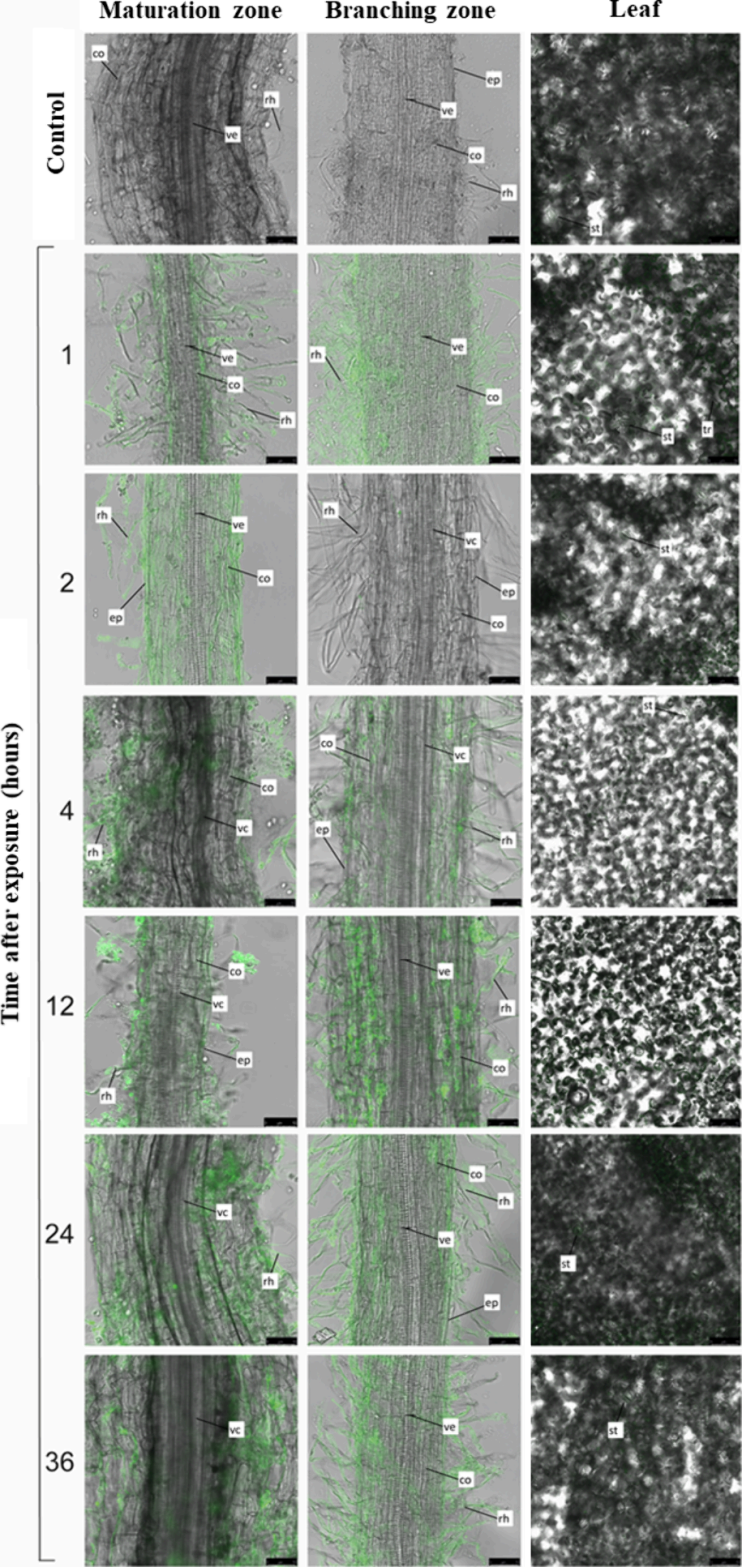
Representative confocal microscopy images of chitosan-coated PCL nanocapsules containing atrazine and labeled with FITC (PCL/CS*f*+ATZ) in *B. pilosa* tissues as a function of exposure time in the hydroponic system (1, 2, 4, 12, 24, and 36 h after exhibition). The figure shows representative images of the root maturation zone, root branching zone, and leaf. Images were obtained at 20× magnification. The negative control refers to plants maintained in distilled water. Stoma (st), trichomes (tr), vascular cylinder (vc), vessel element (ve), cortex (co), epidermis (ep), absorbent hairs (rh). Bars = 50 μm. Data represent means ± standard deviation (*n* = 3).

For PCL/CS*f*+ATZ, images obtained using confocal microscopy showed a higher fluorescence intensity (FI) in the region between the root branching zone and the maturation zone of *B. pilosa* roots (Figure 8), demonstrating that the NCs were absorbed through the primary root structures. However, the intensity is visibly greater near the region of the epidermis and root hairs regardless of the exposure interval (Figure 8 and Figure S3), indicating low penetration.

**Figure 9:**
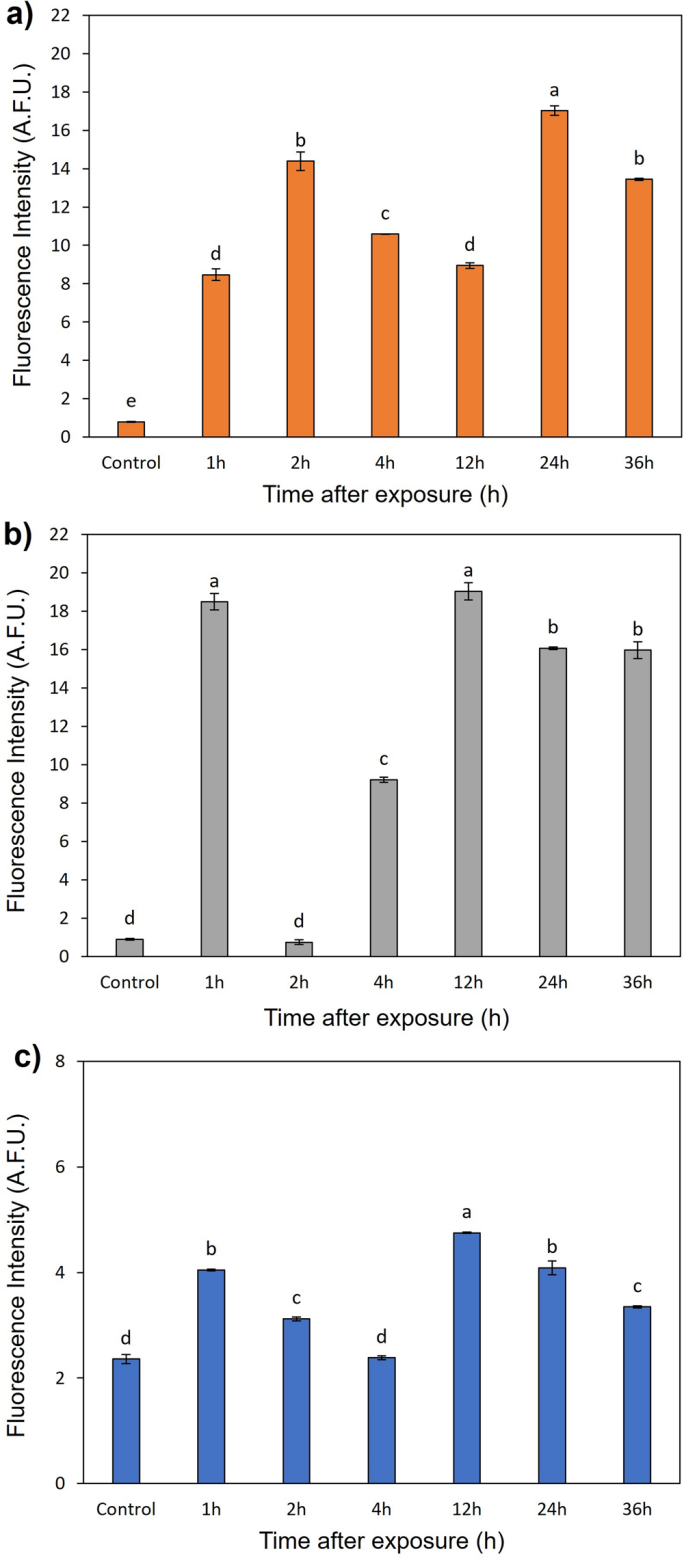
Fluorescence intensity measured as arbitrary fluorescence units (A.F.U.) by confocal microscopy in the (a) root maturation zone, (b) root branching zone, and (c) adaxial surface of the leaf. The negative control refers to plants maintained in distilled water. Letters indicate differences between time intervals (Tukey’s test, *p* ≤ 0.05) in each region. Data represent mean ± standard deviation (*n* = 3).

Through the images, it was also possible to quantify the FI for each region depending on the exposure interval (Figure 9). With a short exposure period (1 h), in the branching zone (Figure 9a) the FI was approximately 2 times higher than in the maturation zone (Figure 9b). In contrast, with exposure of 2 h, there was a drastic drop in the fluorescence signal for this region and a significant increase in the maturation zone compared to the 1 h interval. After prolonged exposure, high FI was still observed in both regions, with similar values after 24 and 36 h of exposure. The low penetration resulted in the accumulation of PCL/CS*f*+ATZ in the root cortex, reaching the vascular cylinder in small quantities, justifying the low FI in leaves (Figure 9c). Despite this, at 1 and 12 h after exposure, the fluorescence signal in the leaves is approximately 2 times higher than in the negative control (Figure 9).

## 4 Discussion

The chitosan coating successfully altered the negative zeta potential of PCL+ATZ NCs to positive, as described by Grillo et al.^39^ This alteration occurs due to the electrostatic interactions of the negative surface of the NCs with the amino radicals present in chitosan molecules.^27,40^ In the current work, the chitosan coating was achieved more successfully, with the encapsulation efficiency exceeding 90%, compared to the value of approximately 65% reported by Grillo et al.^39^ Similar to previous studies, the PCL-based formulations demonstrated stability over time, and the addition of chitosan coating did not alter this stability.^39^ Additionally, release assays illustrated that, even with the coating, the formulations effectively modulated the release of atrazine, exhibiting slower release compared to the commercial product, which could improve the herbicidal effect over time.^36^

Indeed, the PCL/CS+ATZ nanoformulation benefited the postemergence activity of the active ingredient, with gains over the conventional ATZ formulation and the noncoated PCL+ATZ. The gains in PSII activity inhibition of PCL/CS+ATZ over ATZ ranged from 40 to 58% and were reflected in better control of *B. pilosa* plants. Only the dose of 200 g of a.i. ha^–1^ of PCL+ATZ or PCL/CS+ATZ NCs was not sufficient for the control of *B. pilosa* plants, as described in previous studies.^14,41^ However, with 1000 g of a.i. ha^–1^, PCL/CS+ATZ NCs provided excellent plant control, surpassing the results of ATZ at 2000 g of a.i. ha^–1^ and of PCL+ATZ at 1000 g of a.i. ha^–1^.

The recommended atrazine dose for weed control in the early postemergence maize culture (two to four expanded true leaves) is 2000 g of a.i. ha^–1^. Therefore, a significant advancement is achieved with the possibility of a 50% reduction in the active ingredient dose without compromising weed control efficacy, as observed for PCL/CS+ATZ NCs. In greenhouse experiments, with a dose of 1000 g of a.i. ha^–1^ of PCL+ATZ NCs, similar control percentages were obtained as with 2000 g of a.i. ha^–1^ of ATZ in *Digitaria insularis* and *Raphanus raphanistrum* plants.^16,17^ In the study of Wu et al.,^21^ which evaluated the effect of atrazine nanocapsules on lettuce, the authors highlighted that the effects of atrazine and its nanoformulation are different. The authors demonstrated that the nanoformulation had a more prolonged effect. The authors also pointed out that this provides the opportunity to reduce the amount of pesticide needed, either by extending the duration of its effect or by controlling the release time through nanoparticle modifications.

Nanocapsules of positively charged poly(lactic acid) (amino-functionalized, H_2_N) loaded with abamectin showed higher adhesion to leaves (≈58%) compared to negatively charged nanocapsules (≈38%) (carboxylic acid functionalized, CH_3_CO) in cucumber plants.^42^ In both maize (monocotyledon) and cotton (eudicotyledon), positively charged nanoparticles reached the chloroplasts at higher quantities compared to negatively charged nanoparticles.^43^ The epicuticular wax layer found in plants is formed by long-chain hydrocarbons with functional groups such as alcohols, aldehydes, and fatty acids, presenting a negatively charged surface.^44,45^ Possibly, via foliar application, the chitosan coating favored greater interaction and adhesion to the leaf surfaces, as indicated by Grillo et al.^39^ Another consideration is the adhesive characteristic of chitosan-coated nanoparticles,^27,28^ which may also have benefited the adhesion of NCs to the leaves and allowed the active ingredient to reach the action site at higher quantities.

The characteristics of the adaxial face with a high density of trichomes and low stomatal density provide a barrier to the penetration of herbicides into the leaves of *B. pilosa*.^46^ However, the nanoencapsulation of atrazine by PCL (coated or not with chitosan) may have helped overcome these barriers and allowed the active ingredient to reach its site of action more easily, providing greater inhibition of PSII and plant death.

Some studies report that the nanoparticle charge, as well as the composition, has an important effect on the absorption and distribution of particles in the plant, influencing the interaction with functional groups present in the cell wall. Positively charged nanoparticles exhibit greater resistance to root permeability, resulting in greater surface accumulation.^25^ Root hairs are responsible for increasing the contact surface area of the roots.^47^ However, plants have high selectivity regarding the entry of inappropriate compounds into the vascular cylinder, added to which the root epidermis acts as a selective barrier between the external environment and internal plant tissues.^48^

Through the analysis of fluorescence images, it was possible to observe the accumulation of nanocapsules in the roots depending on the exposure time, mainly in the region of the epidermis and root hairs. These results ratified that chitosan nanocapsules have a high adsorption capacity for roots. In a recent study using fluorescently labeled polymers, chitosan nanocapsules also showed a high adsorption capacity for the wheat seed surface, although the involved mechanisms are still unclear.^49^ The detection of a low fluorescence signal related to the nanocapsules in the *B. pilosa* leaves corroborated with the lower amounts of atrazine in this organ and lower inhibition of PSII activity than those observed in the treatment with commercial formulation. Similar results were obtained with zein nanocapsules coated with chitosan, which accumulated in root hairs, epidermis, and cortex, without translocation to the leaves.^36^ Thus, it seems that the greater interaction of PCL/CS+ATZ NCs with the root was detrimental to the translocation to the shoot.

The coating present in PCL/CS+ATZ NCs provided individual interactions with each of the two main entry gates for herbicides in plants. While it proved to be a promising strategy for improving the delivery of the active ingredient to target plants through foliar spray, the greater interaction of NCs with the roots hindered the arrival of the active ingredient at the action site. The biocompatible characteristics and reduction in phytotoxic effects attributed to chitosan coating have the potential to contribute to improving the interaction of NCs with the environment, thus reducing the negative aspects raised about PCL+ATZ. New studies evaluating the action of PCL/CS+ATZ NCs against nontarget organisms remain necessary for this nanosystem.

## 5 Conclusions

Using chitosan to coat the PCL+ATZ NCs nanosystem to change the zeta potential of the NCs was successful. With PCL/CS+ATZ NCs, a new nanosystem for delivering the active ingredient to plants was obtained, with its own release and action patterns. The patterns acquired by the PCL/CS+ATZ nanosystem benefited foliar application, increasing the delivery of active ingredients to plants, the PSII inhibition, and weed control, above the conventional formulation and the PCL+ATZ nanosystem. However, they compromised absorption by the radical system, reducing the arrival of active ingredients at the site of action. Chitosan coating can be an excellent strategy to modify existing NCs systems to facilitate the delivery of active ingredients through the foliar application route.

## Supporting Information Available

Nutritional content used in the hydroponic system solution; infrared spectra and absorption bands for chitosan and fluorescein isocyanate-labeled chitosan; particle size distribution of PCL/CS*f*+ATZ NCs; confocal microscopy images of PCL/CS*f*+ATZ NCs in *B. pilosa* tissues (PDF)

## Abbreviations

a.i.: active ingredient
ATZ: conventional atrazine
FITC: fluorescein isothiocyanate
NCs: nanocapsules
PCL: polycaprolactone
PCL NCs: polycaprolactone nanocapsules
PCL/CS NCs: polycaprolactone nanocapsules coated with chitosan
PCL+ATZ: polycaprolactone nanocapsules containing atrazine
PCL/CS+ATZ: polycaprolactone nanocapsule nanoformulation containing atrazine and coated with chitosan
PCL/CS*f*+ATZ: labeled polycaprolactone nanocapsule nanoformulation containing atrazine and coated with chitosan
PSII: photosystem II
rETR: relative electron transport rate
