# Supplementary material for: Chitosan Coating as a Strategy to Increase Postemergent Herbicidal Efficiency and Alter the Interaction of Nanoatrazine with Bidens pilosa Plants
Source: ACS Appl Mater Interfaces. 2024 Jul 12;17(9):13122–34. doi: 10.1021/acsami.4c03800 (PMC11891830; doi:10.1021/acsami.4c03800)

Supporting Information

Chitosan coating as a strategy to increase post-emergent herbicidal efficiency and alter the interaction of nanoatrazine with *Bidens pilosa* plants

Bruno T. Sousa[1,4ƒ], Lucas B. Carvalho[2ƒ], Ana C. Preisler[1,4], Telma Saraiva-Santos[3], Jhones L. Oliveira[2], Waldiceu A. Verri Jr[3], Giliardi Dalazen[1], Leonardo F. Fraceto[2], Halley Oliveira[4*]*

[1] Department of Agronomy, State University of Londrina (UEL), 86057-970 Londrina, Paraná, Brazil.

[2] Institute of Science and Technology, São Paulo State University (UNESP), 18087-180 Sorocaba, São Paulo, Brazil.

[3] Department of Pathology, State University of Londrina (UEL), 86057-970 Londrina, Paraná, Brazil.

[4] Department of Animal and Plant Biology and Department of Agronomy, State University of Londrina (UEL), 86057-970 Londrina, Paraná, Brazil

***Corresponding Author:** leonardo.fraceto@unesp.br; halley@uel.br

ƒ Authors contributed equally.

Table S1. Final concentration of salts in the nutrient solution used in the experiment with black-jack (*Bidens pilosa*) in hydroponics.

Salt	Concentration
KH_2PO_4	1 mM
$Ca(NO_3)_2.4H_2O$	4 mM
K_2SO_4	2 mM
NH_4NO_3	4 mM
$MgSO_4.7H_2O$	2 mM
H_3BO_3	92,5 µM
$MnCl_2.4H_2O$	18 µM
$ZnCl_2$	1,5 µM
$Na_2MoO_4.2H_2O$	0,56 µM
$CuCl_2.2H_2O$	0,66 µM
$FeSO_4$	100 µM

Figure S1. Infrared spectra for chitosan (CS) and fluorescein isocyanate-labeled chitosan (CS-FITC).

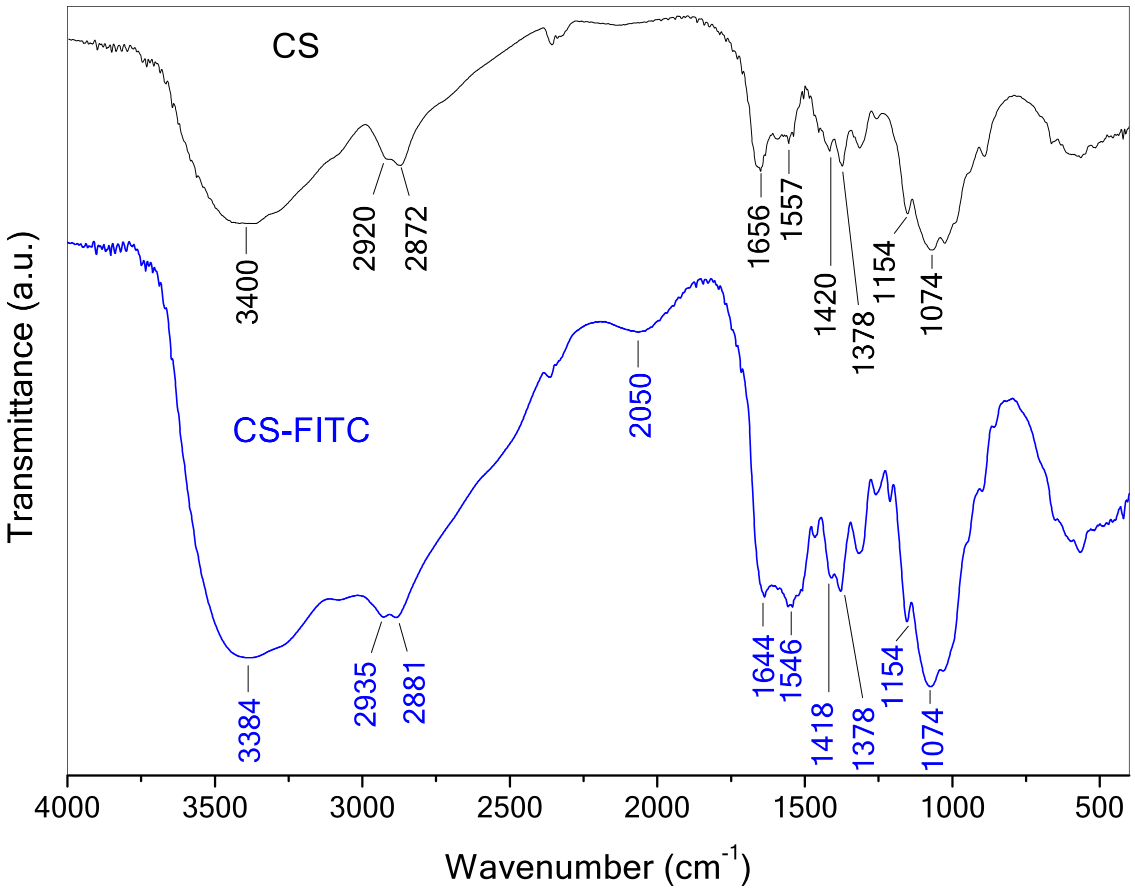

Table S2. Assignments of the absorption bands of the infrared spectrum for chitosan (CS) and chitosan labeled with fluorescein isocyanate (CS-FITC).

Functional group assignment	Absorption bands, wavenumber (cm^{-1})	
	CS	CS-FITC
Axial stretching of O-H and N-H bonds	3400	3384
Axial stretching of C-H bonds	2920 - 2872	2935 - 2881
Stretching of N=C=O bonds		2050
Axial stretching of C=O bonds	1656	1644
Angular deformation of N-H bonds of the amine groups	1557	1546
Coupling of C-N axial stretching and N-H angular deformation	1420 - 1378	1418 - 1378
Stretching of C-O glycosidic bonds	1154	1154
Stretching of C-O-C glycosidic bonds	1074	1074

Figure S2. Particle size distribution PCL/CS*f*+ATZ obtained by nanoparticle tracking technique (NTA).

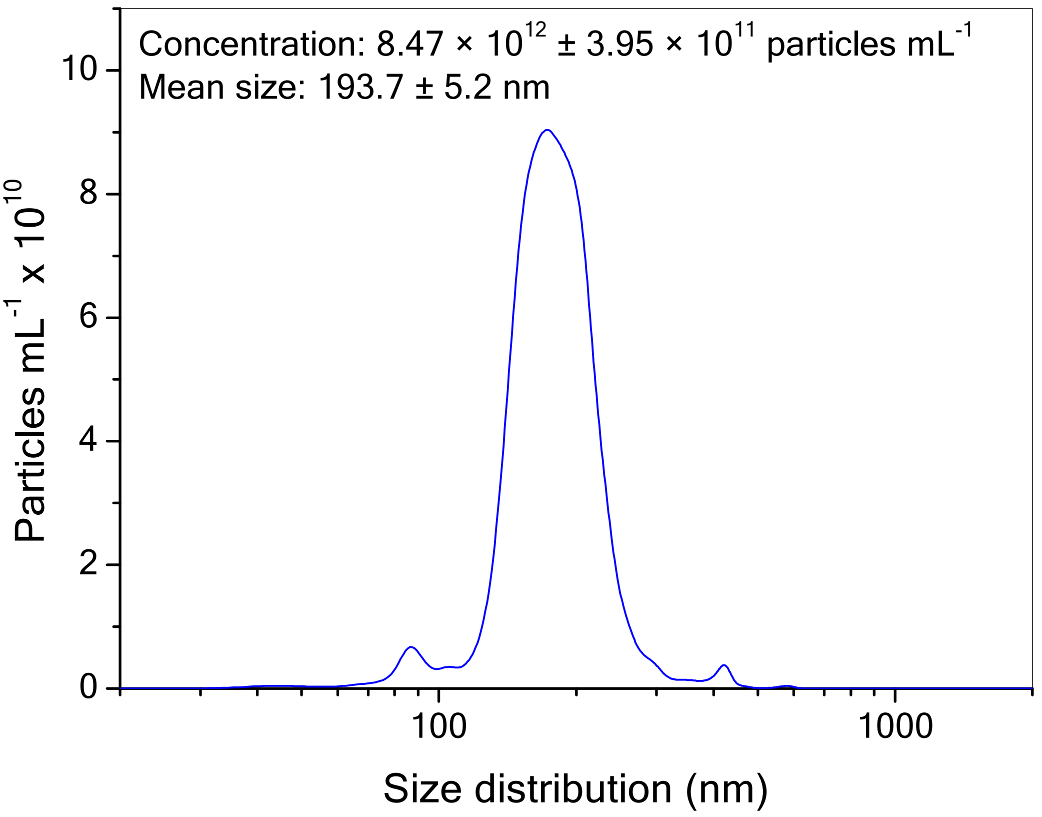

Figure S3. Representative confocal microscopy images of PCL nanoparticles coated with chitosan containing atrazine and labeled with FITC in *B. pilosa* tissues as a function of exposure time in the hydroponic system (1, 2, 4, 12, 24 and 36 h after exposure). The figure shows representative images of the leaf, root in the maturation zone and root in the branching zone. Images were obtained at 20x magnification. Bars = 50 μm

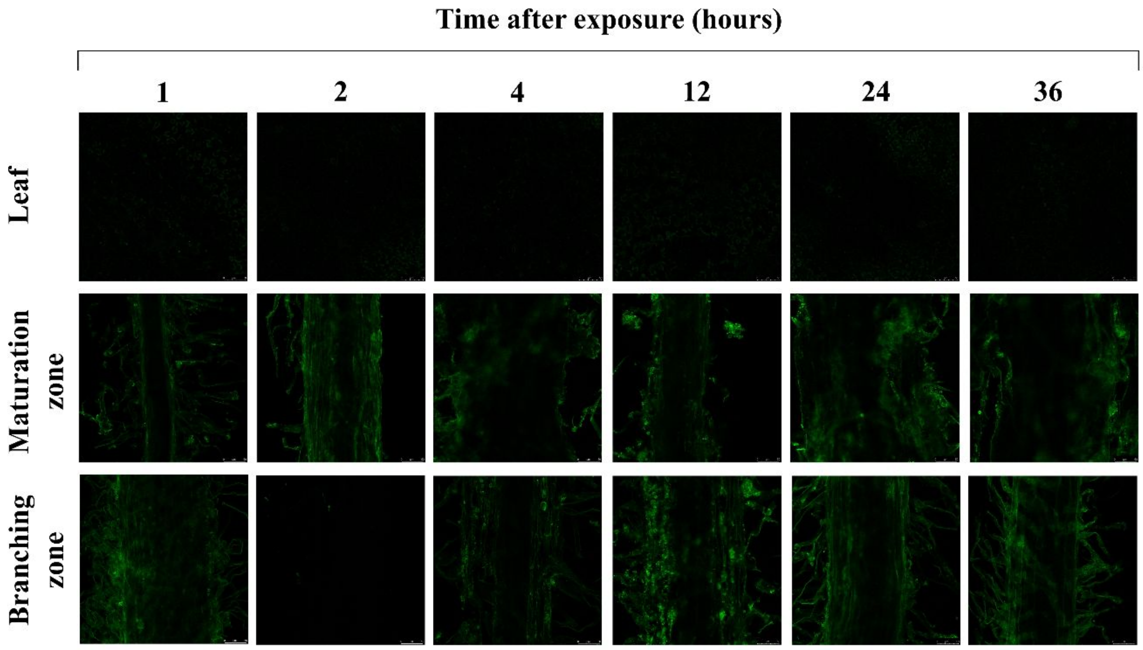

Supplement: Supplementary file 1 — am4c03800_si_001.pdf [file am4c03800_si_001.pdf]
